# Applying normalization process theory to understand implementation of a family violence screening and care model in maternal and child health nursing practice: a mixed method process evaluation of a randomised controlled trial

**DOI:** 10.1186/s13012-015-0230-4

**Published:** 2015-03-28

**Authors:** Leesa Hooker, Rhonda Small, Cathy Humphreys, Kelsey Hegarty, Angela Taft

**Affiliations:** Judith Lumley Centre, La Trobe University, Melbourne, Australia; School of Social Work, University of Melbourne, Melbourne, Australia; Department of General Practice, Primary Care Research Unit, University of Melbourne, Melbourne, Australia

**Keywords:** Normalization process theory, Process evaluation, Intimate partner violence, Maternal and child health nursing, Complex intervention

## Abstract

**Background:**

In Victoria, Australia, Maternal and Child Health (MCH) services deliver primary health care to families with children 0–6 years, focusing on health promotion, parenting support and early intervention. Family violence (FV) has been identified as a major public health concern, with increased prevalence in the child-bearing years. Victorian Government policy recommends routine FV screening of all women attending MCH services. Using Normalization Process Theory (NPT), we aimed to understand the barriers and facilitators of implementing an enhanced screening model into MCH nurse clinical practice.

**Methods:**

NPT informed the process evaluation of a pragmatic, cluster randomised controlled trial in eight MCH nurse teams in metropolitan Melbourne, Victoria, Australia. Using mixed methods (surveys and interviews), we explored the views of MCH nurses, MCH nurse team leaders, FV liaison workers and FV managers on implementation of the model. Quantitative data were analysed by comparing proportionate group differences and change within trial arm over time between interim and impact nurse surveys. Qualitative data were inductively coded, thematically analysed and mapped to NPT constructs (coherence, cognitive participation, collective action and reflexive monitoring) to enhance our understanding of the outcome evaluation.

**Results:**

MCH nurse participation rates for interim and impact surveys were 79% (127/160) and 71% (114/160), respectively. Twenty-three key stakeholder interviews were completed. FV screening work was meaningful and valued by participants; however, the implementation coincided with a significant (government directed) change in clinical practice which impacted on full engagement with the model (coherence and cognitive participation). The use of MCH nurse-designed FV screening/management tools in focussed women’s health consultations and links with FV services enhanced the participants’ work (collective action). Monitoring of FV work (reflexive monitoring) was limited.

**Conclusions:**

The use of theory-based process evaluation helped identify both what inhibited and enhanced intervention effectiveness. Successful implementation of an enhanced FV screening model for MCH nurses occurred in the context of focussed women’s health consultations, with the use of a maternal health and wellbeing checklist and greater collaboration with FV services. Improving links with these services and the ongoing appraisal of nurse work would overcome the barriers identified in this study.

**Electronic supplementary material:**

The online version of this article (doi:10.1186/s13012-015-0230-4) contains supplementary material, which is available to authorized users.

## Background

Intimate partner violence (IPV) is a human rights issue and contributes to serious health, economic and social problems for individual women, children and communities [1-3]. Prevalence rates vary between countries with data from the World Health Organization (WHO) reporting 15–71% of women will experience life time abuse by an intimate partner [1]. In Australia, 17% of women have experienced IPV in their life time especially in the child bearing years [4,5].

Whilst IPV is the most common form of violence towards women, the Australian government uses the term family violence (FV) to encompass IPV when referring to a range of violent behaviours within families [6]. The government defines FV as ‘any violent, threatening, coercive or controlling behaviour that occurs in current or past family, domestic or intimate relationships’ [7]. We adopt the same approach in this paper given the Australian context. As women are predominantly the victims of FV [1], we will refer to men as perpetrators and women and children as victims.

### Screening for family violence

Family violence screening policy and practices have been introduced into many health care settings. We know that screening for FV is acceptable to women [8], increases identification of abuse [9] and safety planning [10] and causes no harm [11]. Screening and supportive care such as advocacy/empowerment interventions may also reduce women’s exposure to subsequent violence, especially in the antenatal period [12,13]. The randomised trial on which this evaluation is based showed a significant and sustained change in clinician behaviour and increase in FV safety planning, 24 months after trial completion (Taft et al., Maternal and child health nurse screening and care for mothers experiencing domestic violence (MOVE): a cluster randomised trial, forthcoming). Many health care professionals continue to face barriers to screening [8,14]. These barriers reduce screening rates and result in practice variation between providers. Consequently, many researchers advocate for case finding rather than screening all women [9,13,15]. More research is needed on the effectiveness and sustainability of FV screening [9] and the context in which change occurs.

### Maternal and child health nursing and family violence

In Victoria Australia, the Maternal and Child Health (MCH) service provides free, universal health care for families with children from birth to school age, seeing more than 95% of new parents [6]. The service aims to maintain health and wellness through health promotion, developmental assessment, early intervention and referral [16]. The MCH service is community-based and staffed by specialist nurse midwives. Similar roles in other countries include public health nurses (USA and Canada) and health visitors (UK) who provide primary health care services to families.

In 2009, the Victorian government introduced a new MCH clinical framework which included mandatory FV screening [6]. The MCH guidelines [6] state that MCH nurses are to ask women routinely about FV at the 4-week postnatal visit and at any other time, if warranted. This policy direction was supported by a one off, 3-hour training session for nurses on FV risk assessment [17]. Rationale for the introduction of routine screening rather than case finding is unclear, but is linked to reducing children’s exposure to FV [18]. More conclusive research and clinical guidance regarding efficacy of FV screening has only recently occurred [9,13].

### MOVE: enhanced MCH nurse care for vulnerable families experiencing violence

Known barriers to screening and recommendations generated from a previous trial (MOSAIC) [19] led to the development of an enhanced model of MCH nursing practice titled MOVE [20]. MOSAIC (a peer support/advocacy intervention) identified that nurses had difficulty identifying abused women, despite receiving extensive FV training [19]. This research highlighted the need for a more nurse-engaged approach. The MOVE model was developed through nurse participatory action research and led to implementation of a range of clinical tools and supportive working practices, with the primary aim of increasing screening, safety planning and referral of abused women in MCH nurse care [20]. Normalization Process Theory (NPT) [21] was used in the development, implementation and evaluation of the MOVE model.

### Normalization process theory

NPT is a socio-behavioural theory focused on the ‘social organization of the work (implementation), of making practices routine elements of everyday life (embedding) and of sustaining embedded practices in their social contexts (integration)’ [21] (p 538). It explores the ‘processes of interventions’ within health services to demonstrate the factors impacting on sustainable changes in practice and is increasingly used in implementation research [22].

### Constructs of NPT

The theory consists of four constructs that describe the organisation of the *action* or *work* performed, and proposes that for a complex intervention (such as FV screening) to become routine everyday practice, we need to consider the following mechanisms - coherence (‘what is the work’), cognitive participation (‘who does the work’), collective action (‘how does the work get done’) and reflexive monitoring (‘how is the work understood’) [21]. These constructs are not linear, but iterative and interrelated.

Drivers of change include individual, organisational, political and economic factors [23]. Process evaluation of complex interventions can identify contextual factors associated with practice change. The aim of this paper is to present the findings of a mixed methods process evaluation of the MOVE cluster randomised trial (Taft et al., forthcoming). To investigate the MCH nursing context surrounding the trial and using NPT; identify the barriers and enablers that have impacted on the successful implementation of the MOVE model.

## Methods

### Study design

A concurrent embedded, mixed method design was used [24], in which quantitative and qualitative data were collected in parallel and analysed using NPT to gain insight into the implementation process. We describe the analysis of key stakeholder interviews and online MCH nurse survey responses that were part of the larger MOVE study (Taft et al., forthcoming) to assess the normalization of FV screening into clinical practice. The MOVE protocol [20] describes a three-stage cluster randomised controlled efficacy trial, based in eight MCH nurse teams in Melbourne, Australia. Process evaluation was aimed at understanding nurse and key stakeholder experiences of the MOVE model in their FV work during the implementation phase. The MOVE protocol provides a detailed description of the MOVE model and the participants’ roles within it [20]. Ethics approval for the entire study was approved by the Human Ethics Committee, La Trobe University (UHEC 08–142) and also by the Victorian Department of Education and Early Childhood Development and the University of Melbourne.

### Setting and participants

The eight MCH nurse teams were located within local government areas from North West Melbourne. The four intervention (IG) team participants comprised MCH nurses, team leaders and nurse mentors. The four comparison (CG) team participants consisted of MCH nurses and team leaders. Other key participants included FV liaison workers and FV managers from two FV services in the region.

### Intervention

Implementation of the MOVE model, which included nurse-designed clinical tools and additional FV liaison support, was carried out over 12 months in the intervention teams [20]. Those with specific roles within the model included nurse mentors and FV liaison workers. These personnel acted as conduits and facilitators between services to ensure adherence to best practice FV work. Nurse mentors (who were MCH nurses) acted as ‘practice champions’ [25], supporting nurses in their everyday FV work (e.g. for secondary consultation), liaising with local FV services and encouraging team discussion about provision of care to women experiencing violence. Two local FV services provided a FV liaison worker. Due to the location and randomisation of MCH nurse teams, one FV liaison worker was allocated three MCH nurse teams, whilst the other FV liaison worker had a one-on-one relationship with the remaining MCH nurse team [20].

Nurses in both arms of the trial received the mandatory, 3-hour FV training session with the introduction of the new government framework [26], and all eight teams had previous FV training during the MOSAIC trial. No additional nurse FV education was provided.

Nurse resources included the MOVE maternal health and wellbeing checklist (with FV screening questions) to be completed by women, a FV clinical practice guideline, clinical pathway and documentation guide to assist with management of women experiencing abuse. The maternal health and wellbeing checklist was completed by women, and this self-completion method allowed women time to reflect on whether to disclose or not. All intervention group nurses were encouraged to provide usual care (FV screening at 4-week visit) and to also use the checklist to screen women at either a specially designated 3-month or the routine 4-month visit, depending on local capacity and preference. The comparison teams continued to provide usual care which included face-to-face screening at 4 weeks and at any other visit if indicated [20].

### Data collection

#### Interim evaluation

All MCH nurses in both arms were asked to complete an online survey at 6 months into the implementation period (survey 1 (S1)). Online survey questions were developed to address NPT constructs and grouped accordingly. Previous research on designing complex interventions assisted data collection tool development [27]. Concepts included nurse attitudes and beliefs about FV, support and safety, skills and knowledge, service system, organisational context and resources and referrals (Table 1, Additional file 1).

**Table 1:** **Interim and impact MCH nurse survey questions relevant to NPT**

**NPT constructs**	
**Coherence**	It is important to screen all women for FV
	MCH nurse interventions can make a difference to the lives of women and children experiencing FV
	I think asking questions about FV at the 4-week consultation is important
	It is important to have a consultation at 3 to 4 months specifically addressing the mother’s health and wellbeing
	I am fulfilling an important community role in discussing FV with my clients
	The FV screening protocol in the new government framework has been very welcome
	I have a good understanding of the issues for women and children experiencing FV
	It is part of my job to have the time to support women experiencing family violence
	I feel uncomfortable when I have to ask all women about FV
	I feel frustrated when women who are abused don’t act on my advice
	I am busy enough without also having to screen all women for FV
	It is the role of the Enhanced nurse team to deal with issues of FV, not mine
**Cognitive participation**	In the past 6 months, I have experienced barriers to asking about FV at 4 weeks
	Overall, what percentage of women have you been able to ask about FV at any time in the past 6 months?
	At what visit are you most likely to ask about FV?
	I ask women who disclose FV about the impact on, and safety of, their children
	I have used the following resources in talking with women about FV
	• Government practice guidelines
	• The Common Risk Assessment Framework (CRAF)
	• Websites
	Intervention group only
	• Nurse mentor
	• FV liaison worker
	• MOVE maternal health and wellbeing checklist
	• MOVE clinical practice guidelines
	• MOVE clinical pathway
**Collective action**	I feel our team of nurses as a group is seriously trying to improve our engagement with clients experiencing FV
**Interactional workability**	There are people in my MCHN team who encourage the team’s FV work
**Relational integration**	I get professional support from my MCH colleagues in FV work
	I feel supported by my team leader in doing this work
	I can turn to my colleagues for emotional support when I am doing this work
	I don’t feel safe visiting women in their homes by myself when there may be FV
	I feel safe in my workplace asking women about FV
	I feel that our work practices mean I feel safe when visiting women at home
**Skill set workability**	I have enough training and skills to ask and respond to women when screening for FV
	I know how to ask women about their safety
	I know how to make a safety plan with women
	I prefer to have a rapport with women before I ask her about FV
	I understand why women don’t leave partners who are abusing them
	If women ask me for help for their abusive partners, I know what information to give women
	I know how to ask women from CALD communities about FV and respond in a culturally sensitive manner
	I know how to ask women from ATSI communities about FV and respond in a culturally sensitive manner
	I can confidently document situations where FV is discussed
	I understand how FV services work
	I am aware of the role of community police in working with women experiencing FV
	I know how to make a referral to Child FIRST
	I know how to make a referral to Child Protection
	I understand the rights of women experiencing FV to access legal, financial and housing support
**Contextual integration**	The CRAF is easy to use
	I have used the CRAF in the past 6 months
	I find FV services responsive when I make a referral
	In the past 6 months I have had difficulty getting appropriate support for women experiencing FV
	I have the time to ask women about FV during the 4 week consultation
	I find Child FIRST services responsive when I make a referral
	I find Child Protection services responsive when I make a referral
	I play my part in addressing the Councils goal of responding to FV in our area
	I feel that the Council does not recognise the importance of the work that we nurses do in relation to FV
**Reflexive monitoring**	We get useful feedback about how well we are doing in our work with FV at team meetings
	Our team has adequate opportunities for supervision with this difficult work

#### Impact evaluation

A slightly modified survey was repeated at 4 months after the trial had ended (survey 2 (S2)). Both surveys were collected using Survey Monkey software [28]. Additional data included open-ended survey responses and completion of 23 face-to-face, semi-structured interviews, averaging 45 minutes. Interviews were completed 9 months post trial, once survey data had been reviewed. These interview questions were also developed according to NPT (Additional file 2). This purposive sample included all intervention MCH nurse mentors and team leaders and comparison team leaders. We also interviewed FV liaison workers and their managers from the two FV services involved.

### Analysis

#### Quantitative data

MCH nurse online survey data were downloaded from the survey database and entered into Excel. Responses were grouped by local government area and trial arm. Responses were then counted and proportions of responses for each questions calculated. These data were compared across trial arms. Differences between trial arm and evaluation time points were analysed using bivariate chi-square tests of independence. An alpha level of 0.05 was used for all statistical tests.

#### Qualitative data

Qualitative content from the online surveys and all interview data were recorded, transcribed, checked and uploaded into the NVivo (9.2) data management system [29].

Understanding NPT and its constructs is a time-consuming and complex process. Directly coding to the constructs of the theory can be a challenge [30], and readers are alerted to the fact that they may need to code sections of their data to multiple constructs simultaneously.

Consequently, we were guided by the NPT tool kit [30] which suggests several ways of using the theory to inform coding and analysis. The Normalization Process Model [31] preceded NPT and consists solely of the sub constructs of *collective action*. We chose to expand the components of this construct in the analysis, as it pragmatically describes the enactment of the work and has been the most widely used aspect of the theory [22].

To help us make sense of the data, we coded inductively (LH) and codes and preliminary themes reviewed by the chief investigator (AT) in regular meetings to ensure reliability of findings. Multiple codes derived from the inductive process were then applied to the four NPT constructs of coherence, cognitive participation, collective action and reflexive monitoring to describe change in practice and contributing factors involved in sustainable FV screening. To aid interpretation, the NPT framework was aligned to derived proposition statements [27,32] for FV work and the MCH nursing context (Table 2). The NPT constructs serve to reframe our inductive coding categories and deepen our analysis. When applied, they highlight the barriers and enablers of sustainable FV screening in MCH nurse practice.

**Table 2:** **Interpretation of NPT applied to MCH nurse practice**

**Proposition statements for FV work**	**NPT constructs and sub components**	**Interpretation in MCH context**
Nurses and stakeholders have a shared understanding and value the FV work.	Coherence (sense making work)	Do MCH nurses think FV is a problem? What practices define FV screening? What is the meaning attributed to screening and FV work? Is there an understanding of the differences between case finding and routine screening? Is the MOVE model easy to describe and distinguish from routine practice?
FV work requires engagement with the model to manage FV in clinical practice.	Cognitive participation (participation work)	How do participants engage in the work? Is it valued? Is there evidence of commitment? Have stakeholders invested time, energy and work into MOVE?
All participants work to operationalize the model within the services. Who is doing the work and the interactions involved?	Collective action (enacting work)	IW - Do MCH nurses think screening at 3–4 months is acceptable? Is it preferred to 4 weeks? Are nurses using the clinical tools and are they worthwhile? How has the use of the clinical tools impacted on the nurse /client interaction?
	• Interactional workability (IW)	
	• Relational integration (RI)	
	• Skill set workability (SSW)	
	• Contextual integration (CI)	
		RI - Is NM knowledge and expertise around FV trusted and understood by nurses? What is the functionality /relationship of the teams and FV services? Are they working, supportive and connected in relation to the work?
		SSW- Do NM feel their role is recognised? Do nurses feel adequately trained and competent to screen women? Do they have confidence in the FV liaison worker to perform secondary consultations?
		CI - How is the FV work funded and supported by local and state government? Have teams successfully negotiated change to incorporate the work?
FV work requires ongoing monitoring of the work.	Reflexive monitoring (appraisal work)	What quality assurance measures are team leaders performing? How is FV work monitored by MCH team leaders? Is there allocated appraisal of the FV work in meetings and with FV services? How are nurses reflecting on their work? Is there evidence of modified practice to improve FV work?

[21,27,32].

## Results

### Participation

Participation in online nurse surveys from both trial arms for the interim and impact surveys was 79% (127/160) and 71% (114/160), respectively. Table 3 shows current hours worked and years as a MCH nurse. The majority of participants worked part time and were an experienced workforce, with ten or more years of clinical experience.

**Table 3:** **Demographic characteristics of all MCH nurse participants in both arms of the MOVE online surveys**

		**All respondents**							
		**INTERIM (127/160) - 79%**				**IMPACT (114/160) - 71%**			
		**MOVE**		**Comparison**		**MOVE**		**Comparison**	
		***n***	**%**	***n***	**%**	***n***	**%**	***n***	**%**
		68	*42.5*	59	*37*	58	*36*	56	*35*
**Work status**									
	MCH full time	21	*16.5*	13	*10.2*	14	*12.3*	11	*9.6*
	MCH part-time	36	*28.3*	36	*28.3*	36	*31.6*	39	*34.2*
	MCH nurse reliever	11	*8.7*	5	*3.9*	5	*4.4*	2	*1.8*
	Enhanced MCH nurse	1	*0.8*	5	*3.9*	3	*2.6*	4	*3.5*
	Other	1	*0.8*	1	*0.8*	0	*0.0*	2	*1.8*
**Years working as MCH nurse**									
	Less than 1 year	1	*0.8*	3	*2.4*	3	*2.6*	6	*5.3*
	1–9 years	28	*22.0*	19	*15.0*	18	*15.8*	18	*15.8*
	10–20 years	18	*14.2*	17	*13.4*	19	*16.7*	19	*16.7*
	Greater than 20 years	21	*16.5*	20	*15.7*	18	*15.8*	13	*11.4*

We present the results of the nurse online surveys (Additional file 1) and interviews according to the NPT constructs (Table 4).

**Table 4:** **Influences on normalisation of FV screening using NPT constructs**

**NPT construct**	**Influencing factors**
**Coherence or** **‘sense making work’**	**Enablers:**
	• Previous FV work
	• Increased discussion of the FV work in team meetings
	**Barriers:**
	• Time of significant change with introduction of KAS framework
	• Some participants misunderstood their role and in several areas, frequent staff changes impacted on implementation
**Cognitive participation or** **‘participation work’**	**Enablers:**
	• Favourable responses of women and the perceived client consciousness raising (primary prevention)
	• Continual team discussions on FV work
	**Barriers:**
	• Lack of privacy to screen in consultations
	• Heavy workloads and competing client demands
	• Limited FV service support
**Collective action or** **‘enacting work’**	
	**Enablers:**
	• Use of the maternal health and wellbeing checklist at 3–4 months enhanced client-nurse interaction and was a ‘good fit’ into the government guidelines
	• Planting a seed by multiple asking points
	• FV liaison worker
	• Adequate knowledge and skill set
	**Barriers:**
	• Poor organisational structure - limited ability to link with other team members or services
	• Low funding and disproportionate allocation of FV liaison worker support to some teams
**Reflexive monitoring or** **‘appraisal work’**	**Enablers:**
	• Clinical supervision allowed for individual reflection
	**Barriers:**
	• Lack of awareness that evaluation and monitoring was required
	• Absence of formal reporting systems in both MCH and FV service

[21,32,42].

### Understanding the MOVE model and FV work: sense making work (coherence)

Over 95% of all nurses felt that FV work was an important part of their role. The majority of clinicians valued the work and had an understanding of the effects of FV on women and children. Coherence improved as nurses continued to implement the model, with the intervention group nurses reporting significantly higher comfort levels with asking about violence on completion of the intervention (S2-IG 65.5%, CG 46.2% (*p* = 0.04)), and this comfort increased over time (S1 46.9% - S2 65.5% (*p* = 0.04)). The importance of screening at the 4-week visit was less for intervention group nurses (S1-IG 44.6%, CG 73.6% (*p* = 0.002) and indicated a nurse preference and understanding of the model’s aims. The importance and inclusion of a specific maternal health and wellbeing consultation at around 3–4 months was well regarded by all nurses in both arms (>92%).

Both groups continued to feel frustrated at times with women who do not act on nursing advice. Less frustration was expressed over time, especially in the comparison group (S1 36.8% - S2 66.7% (*p* = 0.002)). In the interviews, MCH nurses who had previous experience working with abused women, had a greater understanding of management required and were more inclined to embrace the new screening model and practice change.

Some intervention group nurses already had a maternal wellbeing visit which made the MOVE model easy to incorporate into practice. Continual team discussions regarding FV work also facilitated an understanding of the importance of the work.

I think it was just by the more discussion that there was around family violence issues and just becoming more familiar and more confident with your expertise in dealing with these things. (Team leader 3 Intervention Group)

Comprehension of the model increased as MOVE nurses become more proficient at screening, began to use the maternal health and wellbeing checklist and see the favourable responses from women. Nurse consultants involved in the participatory action research had the greatest level of coherence. Those who were uncertain of the model and work involved included relieving or new staff and several nurses confused about their role in the implementation process, e.g. as nurse mentor. The introduction of the MOVE model also coincided with a significant change in MCH practice with the introduction of the new government framework. It was a time when nurses were overloaded with new information and needing to incorporate new additional complex practices into their routines. This may have impacted on the embedding and successful integration of the model.

….at that time some people weren’t very receptive to, you know, anything else at that time as much as they might be on another occasion. (Nurse mentor 3)

### Engaging with the MOVE model: participation work (cognitive participation)

Engagement with the MOVE model was dependant on nurse understanding and the degree of organisational support at local government and FV service level (e.g. funding for the work). Asking about FV increased as nurses became more familiar with the resources and screening practices, with intervention group nurses asking about violence later in the postnatal period, as was recommended (asking at 4 weeks was significantly less in the MOVE teams (S2-IG 62.3%, CG 88.2% (*p* = 0.002)).

The maternal health and wellbeing checklist was used by 96% of intervention group nurses who responded to the survey, and engagement was facilitated due to its ease of use and ‘good fit’ into the existing government framework.

They (nurses) liked the checklist, even to the point where they didn’t … when they didn’t have to use it anymore they decided that they would continue to use it. (Team Leader 4 Intervention Group)

Resource use by intervention nurses increased over time but was low in some areas, especially when engaging with nurse mentors (S1 37.7% - S2 51.9%) or FV liaison workers (S1 26.4% - S2 35.2%) for secondary consultation support and guidance. There was greater use of the MOVE clinical practice guidelines (S1 69.8% - S2 74.1%) and clinical pathway (S1 64.2% - S2 66.7%). Engagement with the work was enhanced by the favourable responses of women to the checklist and the perceived client consciousness raising nature of the work.

Lack of time, privacy and referral sources were the most common reasons for not screening in clinical practice. More than 77% of nurses reported experiencing barriers when asking about violence at the mandatory 4 weeks. Heavy workloads were frequently cited as a barrier to FV work. Although nurses believed it was part of their job to support women experiencing FV, this was logistically challenging in terms of having the time. With the introduction of the government framework, MCH nurses had an increase in structure and specific formats to follow. Screening for FV is only one aspect of nurse work and nurses frequently commented on workloads and their lack of ability to complete all work required within the allocated consultation time. This was especially so if mothers had pressing issues that required immediate attention. This can be a common occurrence in the first few months of parenthood when sleep and feeding issues dominate discussions.

…what they expect us to do in that timeframe (four week visit) was very overwhelming…. and when you have got an issue, it might be a breastfeeding issue, that’s so important to deal with that and sometimes the time to do anything else is gone. (Nurse mentor 3)

The most significant obstacle to engaging in screening was lack of privacy. Nurses and team leaders in both arms spoke of the government policy to screen at 4 weeks being too early as other family members continue to attend consultations with women in the early postnatal period. The MOVE maternal health and wellbeing checklist implemented at 3–4 months partially overcame this obstacle; however, privacy still remained a challenge for FV screening, especially with migrant and refugee women.

FV service allocation varied across the four MCH interventions. Subsequently, the allocation of FV service resources to the MOVE trial was limited, uneven across teams and inadequate to improve the links proposed. The team that had an established relationship with the FV liaison worker appeared to have greater engagement and enhanced work as they achieved the highest screening rates and safety plans (Taft et al., forthcoming).

### Organising change and relationships: enacting work (collective action)

#### Interactional workability - how the complex intervention was operationalised by nurses

The maternal health and wellbeing checklist was highly valued, enhancing the client-nurse interaction and made integration and embedding of FV screening easier for nurses. The checklist was generated by nurse consultants, so it had credibility and was compatible with the government framework. Women were encouraged to self-complete the checklist, following evidence that women prefer this method of asking about abuse [33]. This ‘less direct’ approach to FV screening may have made nurses more comfortable responding rather than asking.

The checklist was great. I think that was very…I know a lot of the nurses found that really helpful, rather than actually having to verbally ask the question. (Nurse Mentor 10)

Asking at the mandated 4 weeks and again with the checklist at 3 or 4 months was seen by some nurses to be beneficial, with nurses feeling that they had ‘planted a seed’ for some women and that asking again (with the checklist) at 3 or 4 months facilitated discussion and trust, with the potential for future disclosure. Although not significant, greater intervention team encouragement (to complete FV work) (S2-IG 83.3%, CG 69.2% (*p* = 0.09)) indicates the level of discussion and enthusiasm for the work. This appeared to foster engagement and implementation of screening.

#### Relational integration -how the work is mediated and understood within social networks

Enhanced collegial discussion about FV and adherence to the safety measures, such as the home visiting policy and procedures introduced in the MOVE model, were important for nurses to feel safe and undertake the FV work. As implementation progressed, intervention nurses felt safer than comparison nurses when attending home visits (S2-IG 82.1%, CG 62.3% (*p* = 0.02)).

Relationships within teams and with FV services varied across the MCH intervention teams. High workloads, time constraints and a lack of nursing staff or relievers in some centres impacted on the organisation of the FV work at times. The nurse mentor role to provide secondary consultation, linkage to FV services and support for other MCH nurses had varied success. Due to time constraints and the often solo nature of MCH practice, most nurses preferred to discuss clinical issues with a nurse friend or co-worker at the time rather than try to contact the designated MOVE nurse mentor, with only 38% of nurses using the nurse mentor role early in the trial. This increased to 52% as time went on. If the nurse was not comfortable speaking and had insufficient time or access to the nurse mentor, then this aspect of the model was lost.

… a lot of them would still go their local, … a neighbouring nurse because that’s who they catch up with more and it was more feasible to catch up, at lunch time or on the phone or something because … we have those networks in our own little area.. (Nurse Mentor 3)

Inadequate FV service collaboration proved a barrier to successful normalization of the model, particularly for teams sharing a FV liaison worker. Nurses working in isolation and with heavy workloads, lack flexibility to attend meetings and make connections with external services. With limited allocation of FV service resources, it was very difficult to promote networking and collaboration.

However, FV work was enhanced for some nurses, especially when there was one-on-one support from the FV liaison worker. This improved collaboration resulted in mutual respect and trust. The work included facilitated linkage with FV services, productive secondary consultations and clinical support, such as joint home visits.

.. I mean I went out and I sat in on a child protection assessment and the nurses trusted me with things like that and rang to do consultations … (FV liaison worker)

It was noted that both sectors felt that the networking that did take place was beneficial to gaining a cultural awareness of the corresponding service. This may be the first step towards improving intersectoral collaboration. Nonetheless, even when teams did have improved connections with FV services, the working relationship was not fully embedded or integrated into their work.

#### Skill set workability - division of labour and distribution/conduct of the work

Nurses felt capable and skilled to address the FV work (S2-IG 85.5%, CG 84.6% (*p* = 0.9)) and complete safety plans (S2-IG 87.3%, CG 82.7% (*p* = 0.5)). Understanding why women do not leave abusive partners increased significantly over time points, but more so in the CG (S1 82.1% - S2 96.2% (*p* = 0.02)). Confidence in asking about FV with migrant and refugee clients (S2 IG 61.8%, CG 59.6% (*p* = 0.82)) and Aboriginal and Torres Strait Islander (ATSI) women (S2 IG 50.9%, CG 51.9% (*p* = 0.92)) was lower than the general population, with privacy and limited knowledge of culturally specific services/resources offered as reasons.

Whilst nurses had received government FV training, many discussed the need for continuing professional development. All team leaders suggested ongoing FV training and skill development.

.. well my concern is that the department has given the training like they’ve ticked off a box, here it is, go out and do it without that ongoing support or updating. (Team Leader 3 CG)

#### Contextual integration - work within the organisational setting

As previously discussed, the organisational structure of MCH and limited resource allocation of FV services meant that nurses’ ability to network with services was restricted.

I think the MCH nurses don’t have a lot of time either, so even going to meetings is difficult and certainly I think we were trying to get some of the nurses to come over here. That was almost impossible. (FV service manager 1)

FV service managers spoke of the overwhelming demand for their services and the need to prioritise resources, with an emphasis on tertiary care.

All nurses reported that external support services were not always responsive to their needs. This included FV and government run child protection services. Less than 50% of nurses agreed that FV services were responsive to referrals (S1 IG 46.4%, CG 41.5% (*p* = 0.61)). This may be the reason for the very low FV referral rates found in the primary research findings. Workloads and time constraints were commonly reported, with nurses needing to adjust work practices to accommodate change. Most nurses felt their local council understood and valued the FV work they did; however, >60% said they did not have enough time allocated during the consultation to address all issues at the 4-week visit.

#### Assessment and monitoring FV work: appraisal work (reflexive monitoring)

Monitoring of work is essential to embedding and maintaining new health care clinical practices. Team feedback about the work (S2-IG 35.2%, CG 21.2% (*p* = 0.11) was slightly higher in the intervention arm, but overall proportions are low. Approximately 65% of intervention nurses reported not getting useful feedback about how well they were doing with the implemented changes to practice. Team leaders reported a lack of awareness that evaluation and monitoring of the FV work was required, despite clear recommendations in the clinical practice guidelines.

I’m not sure how many incidences of disclosure the team have even come across in their work. (MOVE Team leader 1)

Quality assurance measures such as auditing of nurse notes or monitoring of MCH centre responses were not performed as all intervention team leaders reported that they had limited time for these activities. Early in the trial, MOVE nurses reported less opportunity than the comparison group for clinical supervision (S1-IG 48.1%, CG 76.0% (*p* = 0.004)), and this may have had effects on coherence and participation with the work required. By the time the trial had ended, opportunities for individual appraisal had increased (S2-IG 61%, CG 71% (*p* = 0.28)).

## Discussion

Introducing new models of working in health care settings can be challenging [21]. Participants in this study have shown varied responses to implementing and embedding this complex intervention into their practice. It is evident that the MOVE model enhanced many aspects of MCH nurses’ work around FV; however, barriers continue to block integration and normalization. Our findings support evidence that successful implementation of new clinical practices must consider the social, organisational and environmental context and not solely focus on individual behaviour [34,35]. MCH nurse work is operationalised within complex environmental/social systems. Whilst individual practitioner change is necessary, the operational context must also be supportive of implementing new clinical practices.

The following discussion describes which aspects of the trial did and did not work and why, and what is necessary to make improved FV care ‘normal’ and sustainable practice?

### Aspects that facilitated normalization of the MOVE FV model

The most influential aspect of the MOVE intervention model proved to be the maternal health and wellbeing checklist. This tool facilitated *cognitive participation and collective action* especially the *interactional workability* between clinician and client. The checklist was embraced by nurses and complemented the mandatory 4-week screening, which prepared women for the later screening appointment and allowed increased nurse-client dialogue and trust. The less direct nature of asking (using the checklist) appealed to nurses and women, and the self-completion checklist design, moving from physical and emotional health issues towards questions of violence, gave women time to consider their comfort to disclose abuse. This method drew from the evidence around client preference for self-completion screening tools [33].

A multiple asking strategy is supported by the trans theoretical ‘stages of change’ model [36], and is a more women-centred approach allowing women several methods and opportunities to consider whether they wish to discuss FV in this postnatal health care setting. MCH nurses are also going through ‘stages of awareness’ or readiness and the two will only align (ask and disclose) when both are ready. This interaction has mixed success with regard to outcomes (disclosure and referrals), as women are often not ready to tell and the nurse sometimes is not ready to listen. The maternal health and wellbeing checklist helps accelerate this ‘dance of disclosure’ between the two parties [37]. Those teams that included a specific 3-month maternal health visit appeared to make best use of the tools and had improved asking and development of safety plans (Taft et al., forthcoming).

Improved linkage and *relational integration* with FV services also enhanced the FV practices of nurses as collaboration had a positive outcome on the screening and safety plan rate, especially for those teams with best access to FV services (Taft et al., forthcoming). Successful *contextual integration* depends on organisational support, resource allocation and adequate funding. When the FV liaison worker was available to meet with nurses, offer secondary consultations (*relational integration*) and provide a link to wider FV resources and services, nurses felt more supported and comfortable about asking. Other factors that are essential for screening to continue include ensuring safety to perform the work, maintaining regular discussion in team meetings (*coherence*) and formal monitoring of the FV work (*reflexive monitoring*), to ensure the work remains on the agenda.

### Aspects that were detrimental to normalization of the MOVE FV model

There were significant external barriers to screening (*cognitive participation*) and the change process (*collective action*). These included the lack of privacy to screen women, especially at the 4-week visit. Continued partner and family presence in the postnatal setting make this a difficult hurdle to cross as family-centred practice is at the core of MCH nursing. Heavy workloads with rigid routines, and inflexible calendars, make it difficult for MCH nurses to break from set practices. The coinciding introduction of the new government framework and the strict work practices meant that using the screening tools and resources often needed to be balanced against higher client care priorities. Excessive workloads impacted on *coherence* levels and *participation* in the work, with reduced capacity for networking (*relational integration*) and appraisal (*reflexive monitoring*). Organisational support (*contextual integration*) is essential for normalization to occur. Nurse mentors and team leaders in particular had limited time to reflect on and monitor (*reflexive monitoring*) the FV work of nurses and maintain an active discourse and link with FV services. Improvement by MCH nurses to record and monitor FV work is needed to sustain FV screening practices. Individual appraisal through strategies such as MCH nurse clinical supervision may be useful. This lack of structured reflection was detrimental to normalization.

### What is necessary to improve nurse FV work?

The trial findings suggest that the MOVE model significantly enhanced the FV work of MCH nurses (Taft et al., forthcoming). These results may help identify future work needed to promote sustainable FV care in health care settings. Dissemination of appropriate clinical tools that accommodate existing practices, such as the MOVE checklist, clinical guidelines and pathways would support nurses’ FV work and improve clinical practice. The application of these tools at a later, specific maternal wellbeing consultation, where the mother is the only focus of care, was a clear preference of nurses. This additional focused visit allows adequate time and workload allocation to address frequent maternal health concerns.

Ongoing government support and FV training for nurses is essential for sustainable practice in this area, as one off training sessions to implement substantial system change is inadequate [38]. Our survey results indicate that some nurses still feel frustrated with women who do not act on their advice. This lack of understanding and recognition of women’s ‘readiness for change’ indicates that more nurse education is needed in strength-based, women-centred practice and care for abused women and children [39].

### Strengths and limitations

This process evaluation strength includes the combination of a mixed method approach, being embedded in a strong randomised controlled trial design, high participation of nurses in the surveys and the rigorous nature of the analysis using NPT to interpret aspects of practice change and normalization. Nurse responses to screening are consistent with many other studies that report barriers to FV screening in clinical practice [8,14,40]. Limitations include its applicability to the wider postnatal nurse work force, as other nurses may not have the same level of knowledge and skill as reported here, requiring more effort to improve practice [41].

## Conclusion

Implementation of a best practice model (MOVE) of FV screening and care in MCH services has been formatively evaluated, using NPT. Theory-based process evaluation of complex interventions assisted in explaining what worked and what did not work in MOVE and in identifying processes likely to enhance sustainability. Implementing interventions in complex, multi-tiered systems such as MCH services is challenging. Findings suggest that the enhanced FV work of MOVE was meaningful and valued by participants, even though implementation coincided with significant government-introduced practice changes which may have impaired engagement with the model (*coherence and cognitive participation*). The use of MCH nurse-designed FV screening resources and supportive links with FV services enhanced the participants’ work (*collective action*). Monitoring and ongoing management of FV screening needs improvement (*reflexive monitoring*). Identification of these factors provides a greater understanding of what is required to enhance the future FV work of community-based nurses and improve the support and safety of women and their children.

## Additional files

Additional file 1:
**MOVE Maternal and Child Health nurses’ online survey analysis using NPT.**
Additional file 2:
**MOVE impact evaluation interview questions.**

## Abbreviations

ATSI: Aboriginal and Torres Strait Islander
CG: comparison group
CI: contextual integration
CRAF: common risk assessment framework
EFT: equivalent full time
FV: family violence
IG: intervention group
IPV: intimate partner violence
IW: interactional workability
KAS: key age and stage
MCH: maternal and child health
MOVE: improving maternal and child health care for vulnerable mothers project
NM: nurse mentor
NPT: normalization process theory
RI: relational integration
SSW: skill set workability
