# Supplementary material for: Applying normalization process theory to understand implementation of a family violence screening and care model in maternal and child health nursing practice: a mixed method process evaluation of a randomised controlled trial
Source: Implement Sci. 2015 Mar 28;10:39. doi: 10.1186/s13012-015-0230-4 (PMC4379540; doi:10.1186/s13012-015-0230-4)
Supplement: Additional file 1: — MOVE Maternal and Child Health nurses’ online survey analysis using NPT. [file 13012_2015_230_MOESM1_ESM.pdf]

MOVE Maternal and Child Health nurses' online survey analysis using NPT

Coherence

	**Interim Survey (S1) Results**					**Impact Survey (S2) Results**						**Difference**			
	MOVE Group (IG)	%	Comparison Group (CG)	%	p value		MOVE Group (IG)	%	Comparison Group (CG)	%	p value	Overall change IG-IG %	p value	Overall change CG-CG %	p value
It is important to screen all women for FV (64/57)	61 (agree)	*95.3*	55	*96.5*	0.74	NA									
MCH nurse interventions can make a difference to the lives of women and children experiencing FV (64/57)	61(agree)	*95.3*	53	*93*	0.58	NA									
**I think asking questions about FV at the 4 week consultation is important (56/53)**	**25**(agree)	***44.6***	**39**	***73.6***	**0.002**	NA									
It is important to have a consultation at 3-4 months specifically addressing the mother's health and well-being (56/53)	47(agree)	*83.9*	46	*86.8*	0.67	(55/52)	51	*92.7*	48	*92.3*	0.93	↑8.8	0.15	↑5.5	0.36
I am fulfilling an important community role in discussing FV with my clients (52/50)	50(agree)	*96.1*	48	*96*	0.97	NA									
The FV screening protocol in the new government framework has been very welcome (64/57)	45(agree)	*70.3*	43	*75.4*	0.53	NA									
I have a good understanding of the issues for women and children experiencing FV (60/56)	51(agree)	*85*	45	*80.4*	0.51	(55/52)	48	*87.3*	47	*90.4*	0.61	↑2.3	0.73	↑10.0	0.14

	Interim Survey (S1) Results					Impact Survey (S2) Results						Difference			
	MOVE Group (IG)	%	Comparison Group (CG)	%	p value		MOVE Group (IG)	%	Comparison Group (CG)	%	p value	Overall change IG-IG %	p value	Overall change CG-CG %	p value
It is part of my job to have the time to support women experiencing family violence (64/57)	55(agree)	*85.9*	50	*87.7*	0.77	(55/52)	52	*94.5*	49	*94.2*	0.94	↑8.6	0.12	↑6.5	0.24
**I feel uncomfortable when I have to ask all women about FV (64/57)**	30 (disagree)	*46.9*	30	*52.6*	0.53	(55/52)	36	*65.5*	24	*46.2*	**0.04**	↑ 18.6	**0.04**	↓ 6.4	0.5
**I feel frustrated when women who are abused don't act on my advice (64/57)**	30(disagree)	*46.9*	21	*36.8*	0.26	(55/51)	29	*52.7*	34	*66.7*	0.14	↑5.8	0.52	↑29.9	**0.002**
I am busy enough without also having to screen all women for FV (64/57)	50(disagree)	*78.1*	50	*87.7*	0.16	(51/54)	42	*82.4*	47	*87*	0.5	↑4.3	0.57	↓0.7	0.91
It is the role of the Enhanced nurse team to deal with issues of FV, not mine (52/50)	45(disagree)	*86.5*	38	*76*	0.17	(53/52)	45	*84.9*	44	*84.6*	0.97	↑1.6	0.81	↑8.6	0.27

Cognitive Participation

	Interim Survey (S1) Results					Impact Survey (S2) Results						Difference			
	MOVE Group (IG)	%	Comparison Group (CG)	%	p value		MOVE Group (IG)	%	Comparison Group (CG)	%	p value	Overall change IG-IG %	p value	Overall change CG-CG %	p value
In the past 6 months I have experienced barriers to asking about FV at 4 weeks	NA					Yes (54/52)	48	*88.9*	40	*76.9*	0.1				
Overall, what percentage of women have you been able to ask about FV at any time in the past 6 months?	NA					> 50% (55/52)	45	*81.8*	44	*84.6*	0.7				
**At what visit are you most likely to ask about FV?**	NA					≤ 4weeks (53/51)	33	*62.3*	45	*88.2*	**0.002**				
I ask women who disclose FV about the impact on, and safety of, their children (60/56)	50	83.3	49	87.5	0.53	Yes (55/52)	50	*90.9*	47	*90.4*	0.93	↑7.6	0.23	↑2.9	0.63
I have used the following resources in talking with women about FV (53/51)						(54/51)									
Government practice guidelines	45	*84.9*	47	*92.1*	0.25		51	*94.4*	48	*94.1*	0.94	↑9.5	0.1	↑2	0.7
The Common Risk Assessment Framework (CRAF)	28	*52.8*	30	*58.8*	0.54		35	*64.8*	34	*66.7*	0.84	↑12	0.21	↑7.9	0.41
Websites	6	*11.3*	6	*11.8*	0.94		7	*13*	11	*21.6*	0.24	↑1.7	0.79	↑9.8	0.18
Nurse mentor-**IG only**	20	*37.7*					28	*51.9*				↑14.2	0.14		
FV Liaison Worker	14	*26.4*					19	*35.2*				↑8.8	0.33		
MOVE MWB checklist	53	*100*					52	*96.3*				↓3.7	0.5*		
MOVE Clinical practice guidelines	37	*69.8*					40	*74.1*				↑4.3	0.62		
MOVE clinical pathway	34	*64.2*					36	*66.7*				↑2.5	0.78		

Collective Action

	Interim Survey (S1) Results					Impact Survey (S2) Results						Difference			
	MOVE Group (IG)	%	Comparison Group (CG)	%	p value	Impact survey responses	MOVE Group (IG)	%	Comparison Group (CG)	%	p value	Overall change IG-IG %	p value	Overall change CG-CG %	p value
**Interactional workability**															
I feel our team of nurses as a group is seriously trying to improve our engagement with clients experiencing FV (52/50)	44 (agree)	*84.6*	40	*80*	0.54	(54/52)	47	*87*	41	*78.8*	0.26	↑2.4	0.72	↓1.2	0.89
There are people in my MCHN team who encourage the team's FV work	NA					(54/52)	45	*83.3*	36	*69.2*	0.09				
**Relational integration**															
**I get professional support from my MCH colleagues in FV work (64/56)**	53 (agree)	*82.8*	40	*71.4*	0.14	(55/53)	47	*85.5*	46	*86.8*	0.84	↑2.7	0.69	↑15.4	**0.05**
I feel supported by my team leader in doing this work (52/50)	36 (agree)	*69.2*	30	*60*	0.33	(54/52)	35	*64.8*	38	*73.1*	0.36	↓4.4	0.63	↑13.1	0.16
I can turn to my colleagues for emotional support when I am doing this work (64/56)	57 (agree)	*89.1*	45	*80.3*	0.18	(55/53)	46	*83.6*	44	*83*	0.93	↓5.5	0.39	↑2.7	0.72
I **don't** feel safe visiting women in their homes by myself when there may be FV (64/56)	44 (agree)	*68.8*	32	*57.1*	0.19	NA									
**I feel safe in my workplace asking women about FV (64/56)**	53 (agree)	*82.8*	53	*94.6*	**0.04**	(55/53)	51	*92.7*	50	*94.3*	0.73	↑9.9	0.1	↓0.3	0.94

	Interim Survey (S1) Results					Impact Survey (S2) Results						Difference			
	MOVE Group (IG)	%	Comparison Group (CG)	%	p value	Impact survey responses	MOVE Group (IG)	%	Comparison Group (CG)	%	p value	Overall change IG-IG %	p value	Overall change CG-CG %	p value
**I feel that our work practices mean I feel safe when visiting women at home**	NA					Agree (56/53)	46	*82.1*	33	*62.3*	**0.02**				
**Skill set workability**															
I have enough training and skills to ask and respond to women when screening for FV (60/56)	45 (agree)	*75*	43	*76.8*	0.82	(55/52)	47	*85.5*	44	*84.6*	0.9	↑10.5	0.16	↑7.8	0.3
I know how to ask women about their safety (60/56)	53 (agree)	*88.3*	54	*96.4*	0.1	NA									
I know how to make a safety plan with women (60/56)	50 (agree)	*83.3*	44	*78.6*	0.51	(55/52)	48	*87.3*	43	*82.7*	0.5	↓4.0	0.55	↑4.1	0.59
I prefer to have a rapport with women before I ask her about FV (60/56)	43 (agree)	*71.7*	35	*62.5*	0.29	NA									
**I understand why women don't leave partners who are abusing them (60/56)**	52 (agree)	*86.7*	46	*82.1*	0.5	(55/52)	50	*90.9*	50	*96.2*	0.27	↑4.2	0.47	↑14.1	**0.02**
If women ask me for help for their abusive partners, I know what information to give women (60/56)	54 (agree)	*90*	48	*85.7*	0.48	(55/52)	51	*92.7*	47	*90.4*	0.66	↑2.7	0.6	↑4.7	0.46
I know how to ask women from CALD communities about FV and respond in a culturally sensitive manner (60/56)	39 (agree)	*65*	38	*67.9*	0.74	(55/52)	34	*61.8*	31	*59.6*	0.82	↓3.2	0.72	↓8.3	0.37
I know how to ask women from ATSI communities about FV and respond in a culturally sensitive manner (60/56)	27 (agree)	*45*	34	*60.7*	0.09	(55/52)	28	*50.9*	27	*51.9*	0.92	↑5.9	0.53	↓8.8	0.36

	Interim Survey (S1) Results					Impact Survey (S2) Results						Difference			
	MOVE Group (IG)	%	Comparison Group (CG)	%	p value	Impact survey responses	MOVE Group (IG)	%	Comparison Group (CG)	%	p value	Overall change IG-IG %	p value	Overall change CG-CG %	p value
I can confidently document situations where FV is discussed (60/56)	52 (agree)	*86.7*	46	*82.1*	0.5	(55/52)	50	*90.9*	43	*82.7*	0.21	↑4.2	0.47	↑0.6	0.94
I understand how FV services work (56/53)	36 (agree)	*64.3*	32	*60.4*	0.67	(55/52)	37	*67.3*	36	*69.2*	0.83	↑3.0	0.74	↑8.8	0.34
I am aware of the role of community police in working with women experiencing FV (56/53)	37 (agree)	*66.1*	37	*69.8*	0.68	(55/52)	39	*70.9*	35	*67.3*	0.69	↑4.8	0.58	↓2.5	0.78
I know how to make a referral to Child FIRST (60/56)	54 (agree)	*90*	51	*91.1*	0.84	NA									
I know how to make a referral to Child Protection (60/56)	55 (agree)	*91.7*	54	*96.4*	0.28	NA									
I understand the rights of women experiencing FV to access legal, financial and housing support (56/53)	48 (agree)	*85.7*	46	*86.8*	0.87	NA									
**Contextual integration**															
The CRAF is easy to use (56/53)	32 (agree)	*57.1*	29	*54.7*	0.8	NA									
I have used the CRAF in the past 6 months (56/53)	23 (agree)	*41.1*	23	*43.4*	0.81	NA									
I find FV services responsive when I make a referral (56/53)	26 (agree)	*46.4*	22	*41.5*	0.61	NA									
In the past 6 months I have had difficulty getting appropriate support for women experiencing FV	NA					Yes (55/49)	11	*20*	8	*16.3*	0.63				

	Interim Survey (S1) Results					Impact Survey (S2) Results						Difference			
	MOVE Group (IG)	%	Comparison Group (CG)	%	p value	Impact survey responses	MOVE Group (IG)	%	Comparison Group (CG)	%	p value	Overall change IG-IG %	p value	Overall change CG-CG %	p value
I have the time to ask women about FV during the 4 week consultation (56/53)	22 (agree)	*39.3*	28	*52.8*	0.16	NA									
**I find Child FIRST services responsive when I make a referral (56/53)**	14 (agree)	*25*	25	*47.2*	**0.02**	NA									
I find Child Protection services responsive when I make a referral (56/53)	19 (agree)	*33.9*	27	*50.9*	0.07	NA									
I play my part in addressing the Councils goal of responding to FV in our area (52/50)	45 (agree)	*86.5*	41	*82*	0.53	(53/52)	45	*84.9*	43	*82.7*	0.76	↓1.6	0.81	↑0.7	0.93
I feel that the Council does not recognise the importance of the work that we nurses do in relation to FV (52/50)	15 (agree)	*28.8*	15	*30*	0.9	(53/52)	15	*28.3*	19	*36.5*	0.37	↓0.5	0.95	↑6.5	0.48

Reflexive monitoring

	Interim Survey (S1) Results					Impact Survey (S2) Results						Difference			
	MOVE Group (IG)	%	Comparison Group (CG)	%	p value		MOVE Group (IG)	%	Comparison Group (CG)	%	p value	Overall change IG-IG % difference	p value	Overall change CG-CG % difference	p value
We get useful feedback about how well we are doing in our work with FV at team meetings	NA					Agree (54/52)	19	35.2	11	21.2	0.11				
**Our team has adequate opportunities for supervision with this difficult work (52/50)**	25(agree)	*48.1*	38	*76*	**0.004**	(54/52)	33	*61.1*	37	*71.2*	0.28	↑13	0.18	↓4.8	0.58

CALD-Culturally and Linguistically Diverse

Child FIRST – Family Information, Referral and Support Teams

NA - Question not asked at this time point

Significance at $p < 0.05$ * Fisher's exact test
