# Supplementary material for: Applying normalization process theory to understand implementation of a family violence screening and care model in maternal and child health nursing practice: a mixed method process evaluation of a randomised controlled trial
Source: Implement Sci. 2015 Mar 28;10:39. doi: 10.1186/s13012-015-0230-4 (PMC4379540; doi:10.1186/s13012-015-0230-4)
Supplement: Additional file 2: — MOVE impact evaluation interview questions. [file 13012_2015_230_MOESM2_ESM.pdf]

MOVE IMPACT EVALUATION INTERVIEW QUESTIONS

NURSE MENTORS

1. Can you describe how useful you have found the new Key Ages and Stages Framework in terms of the policy to ask about family violence at the 4 week appointment?
- How useful have you found the resources provided for KAS (CRAF manual, training, computer prompt)

2. Please describe in what ways, if any, you feel the MOVE model (prompt: mentor, FVLW clinical guidelines, clinical pathway, checklist,) has interacted with KAS?
- What was the most important element?

3. What is your perception of how MOVE was received and acted upon by the nurses in your team?
- What issues impeded its 'take up'?
- What promoted/encouraged its 'take up'?

4. In your role as Nurse Mentor in what ways were you able to encourage the good practice of your team's family violence work? (Probe - FV discussions at team meetings, training of new nurses, provision of FV resources, OH&S policies)
- What issues impeded your role as Nurse Mentor?
- What factors/circumstances aided your role as Nurse Mentor?
- How successful do you feel you were in this role?

5. Part of the MOVE best practice model was the efforts to strengthen links with Family Violence and other community services through the Family Violence Liaison Worker.
- How has this worked in your council?
- Were you able in your role as Nurse Mentor to establish liaison with the Family Violence Liaison worker? If yes how, if not, why?

6. In the MOVE Clinical Guidelines it was outlined that the Nurse Mentor's role included maintaining and distributing information about CALD and ATSI specific agencies. Were you able to do this? Can you tell us more about this?

7. Do you think there was benefit in formalising the role of nurse mentor in relation to family violence work within the team? Why?

8. What elements of MOVE, if any, would you like to see sustained?
- How do you think that could be achieved?

9. How did Council contribute to the team's family violence work/MOVE Model?

INTERVENTION GROUP TEAM LEADERS

1. Can you describe for me how you have found the new Key Ages and Stages Framework in terms of assisting the Family Violence work of your team?

2. Please describe in what ways, if any, you feel the MOVE model (prompt: mentor, FVLW clinical guidelines, clinical pathway, checklist,) has interacted with the KAS?

3. What is your perception of how MOVE was received and acted upon by the nurses in your team? What issues impeded it's 'take up' and what promoted/encouraged it's 'take up'?

4. Can you tell me about the issues that have been raised at your team meetings around family violence?
- What issues have been problematic?
- What have been positive?

5. What was your opinion of formalising the role of nurse mentor in relation to family violence work within the team?
- How did you see this role?
- What if any, was its value to your team?

6. What was your opinion of having a Family Violence Liaison Worker linked to your team? Why?
- How did it work for your team? (Strengths /Weaknesses)

7. What was your opinion of the Maternal Health & Wellbeing Checklist being given to each woman to fill in?

8. In your role as Team Leader, in what ways were you able to encourage the use of the MOVE model in your team? Prompts:
- FV discussions at team meetings,
- Opportunities for nurses to rehearse scenarios and questions,
- Training of new nurses,
- OH&S policies
- Any others?

9. In the MOVE Clinical Guidelines there was an emphasis on the Team Leader's role in quality assurance in relation to their team's Family Violence work. Were you able to implement any quality assurance of your team's Family Violence work? What were you able to do? Prompts:
- Audit of notes,
- Allocation of time at team meetings to discuss team performance/raise issues relating to FV,
- Provision of educational updates to FV practice, knowledge and skills,
- Training of new nurses,
- Ensuring OH&S policies address safety issues for nurses working with families,
- Monitoring time management of FV,
- Undertake random surveys of clients to evaluate satisfaction with service – esp FV support

10. Have there been any issues related to the MOVE Project in terms of policy and procedure at either team level or council level?

11. How do you feel the work of the maternal and child health nurse team in relation to family violence is recognised by your council?

12. What elements of MOVE, if any, would you like to see sustained within your team? How can that be achieved?
- What barriers are there to that happening?

13. How you feel the work of the maternal and child health nurse team in relation to family violence is supported by the DEECD?

14. How did Council contribute to the team's family violence work/MOVE Model?

<u>AND IF ALSO A NURSE MENTOR PLEASE ASK:</u>

15. In your role as Nurse Mentor in what ways were you able to encourage the good practice of your team's family violence work? (Probe - FV discussions at team meetings, training of new nurses, provision of FV resources, OH&S policies)
- What issues impeded your role as Nurse Mentor?
- What factors/circumstances aided your role as Nurse Mentor?
- How successful do you feel you were in this role?

16. Part of the MOVE best practice model was the efforts to strengthen links with Family Violence and other community services through the Family Violence Liaison Worker.
- How has this worked in your council?
- Were you able in your role as Nurse Mentor to establish liaison with the Family Violence Liaison worker? If yes how, if not, why?

17. In the MOVE Clinical Guidelines it was outlined that the Nurse Mentor's role included maintaining and distributing information about CALD and ATSI specific agencies. Were you able to do this? Can you tell us more about this?

18. Do you think there was benefit in formalising the role of nurse mentor in relation to family violence work within the team? Why?

COMPARISON GROUP TEAM LEADERS

1. Can you describe for me how you have found the new Key Ages and Stages Framework in terms of assisting the Family Violence work of your team?

2. Can you tell me about the issues that have been raised at your team meetings around family violence? What issues have been problematic? What have been positive?

3. Do you have any nurses in your team who act as resources for the other nurses in your team in relation to family violence? Can you tell me how this works?

4. Does your team have established links with community family violence agencies? How is this encouraged or formalised?
- With family violence workers?
- With community police?
- With ATSI/CALD family violence services?

5. In your role as Team Leader in what ways do you encourage the family violence work of your team? Prompts:
- FV discussions at team meetings,
- Opportunities for nurses to rehearse scenarios and questions,
- Training of new nurses,
- Occupational Health & Safety policies

6. Are there any quality assurance measures in place for the family violence work of your team? Can you describe this for me?

7. What strategies will you need to sustain the family violence work of your team?

8. How do you feel the work of the maternal and child health nurse team in relation to family violence is recognised by your council?

9. How you feel the work of the maternal and child health nurse team in relation to family violence is supported by the DEECD?

FAMILY VIOLENCE LIAISON WORKERS

1. What was your experience of being a Family Violence Liaison Worker in MOVE?
- What were you able to achieve in this role?
- What issues impeded your role as FVLW?
- What factors/circumstances aided your role as FVLW?

2. What liaison were you able to provide to the team/s you were linked to?
- Attend MCH team meetings, advise nurses of FV resources/referral pathways, facilitate discussions of issues related to FV, organise tour of FV service, educate staff at FV service on role of MCH nurses, provide feedback to MCH service on referral rates & any issues arising.

3. In your role as FVLW, in what ways were you able to promote links between the MCH team/s and other services such as local police/legal services/ATSI & CALD agencies?

FAMILY VIOLENCE MANAGERS

1. What do you think has been the outcome of the liaison between your FVLW and the MCH teams involved in MOVE?

- What positive outcomes do you think arose from this liaison?
- What issues do you think impeded the liaison?

2. What has the impact of being involved in MOVE been for your Agency?

3. Would you like to see the relationship with the MCH nurse teams continue?
- If yes, what would it take to make it sustainable?
- If no, can you tell me about that?
